# Race, intelligence and genetics: colonialism in the era of neurotechnology

**DOI:** 10.3389/fpubh.2025.1737069

**Published:** 2026-01-12

**Authors:** Monique Pyrrho

**Affiliations:** Institute of Biological Sciences, University of Brasilia, Brasilia, Brazil

**Keywords:** neurotechnology, neuroenhancement, colonialism, racism, biological determinism, race, intelligence

## Abstract

Unequal access is not the only or even the main ethical challenge concerning neurotechnological advancements in the Global South. Epistemic sanctioned discourses on cognitive abilities are powerful and have been used to subdue and marginalize people. Aside from better understanding and preventing exploitative scientific practices, further investigating the historical relationship between colonialism and science is a necessary step to avoid reproducing structural injustice on ethical and legal frameworks to neurotechnology. In this sense, this paper discusses a conceptual matter on neurotechnology that demands further attention, namely the role that intelligence discursively plays in colonialism and racism.

## 1 Introduction

Unequal access to the benefits of science is a ubiquitous topic in ethics of neurotechnology, but the reasons or possible ways to mitigate it are not accordingly addressed. Dehumanization is the engine of colonial matrix of power, and the concept of intelligence historically plays a major part in it. Nevertheless, by only mentioning this unfairness, ethical discourses at once manage to set themselves apart from the mere-profit-driven practices, while reaffirming the credo that increasing amounts of technology is what is lacking to achieve a more just society. Moreover, waiting time-related lowering costs of technology is not tackling injustice.

Additionally, exploitative praxis, such as conducting parachute science, taking specimens and appropriating knowledge from native peoples in the Global South without due recognition and retribution are examples of colonialism in science. Research and development funding is necessary to reduce disease burden and other problems in low-income countries and marginalized populations, but those populations’ needs are time and time again neglected by scientific agenda. Local expertise participation is often seen as a means to get access to the ground. Scientific collaboration between the Global North and South reflects disparities in power and unevenly distributes scientific and economic results. These colonial practices in science reinforce inequality (1–3).

Further exploring the historical relationship between colonialism and science is a necessary step to avoid reproducing structural injustice in ethical and legal frameworks of neurotechnology. Aside from better understanding and preventing the above-mentioned aspects, there is a conceptual matter regarding neurotechnology that demands further attention, and it has to do with the role that intelligence discursively plays in colonialism and racism.

Colonial and eugenics legacies are still more influential than we “dare to admit” (4). And historically, the prerogative of enunciating what is rational or normal, intellectually inferior and superior has justified the destitution and elimination of every insubordinate way of living and knowing (5). Claiming universality and humanistic purposes, Western thought produced definitions of rationality, intelligence, mental health, and of functional, productive and enhanced minds. These are scientifically sanctioned discourses, but they are neither neutral nor universal. These same conceptions and their resulting ways of ranking people have historically been used to validate some forms of distress, while silencing and oppressing others. Knowledge about mental health is power and it has been used to subdue and marginalize people (6). Given that part of neurotechnoscience reissues the mission of enhancing intelligence for humanistic reasons, there are reasons to be concerned. There are also opportunities to wonder whether and how a non-colonial approach to neurotechnology may exist.

The present paper explores mechanisms that perpetrate unequal access to the benefits of science, highlighting scientific constructs and discourses that contribute to naturalizing injustice. The contextualization of the recent controversy on intelligence and race may offer some insights on paths to advance non-colonial ethical and legal approaches to neurotechnologies.

## 2 Biological determinism and colonialism

Ranking groups of people according to cognitive abilities enabled eugenics and is a key factor in racism, sexism and classism. The association between intelligence, racism and domination dates to colonialization (7).

Colonialism is based on combined strategies of hierarchization, dehumanization and exploitation. Post-colonial and decolonial studies have different geographical and theoretical backgrounds on the subject, but scholars identified the centrality of epistemic dimension to colonialism in common from the beginning (8, 9). In this context, knowledge, as a manifestation of superior intelligence, justified the *mission civilisatrice*, but it was technology which provided the means for mastering nature, including colonized bodies. Accordingly, the term technological colonialism nowadays refers to resourcing technoscientific concepts and tools to reinforce colonial power dynamics (6).

Biological determinism and the relationship between race and intelligence played a major role in the process. Müller-Wille (10) observes that the concept of race is frequently told to have three different moments throughout modern history. First, Enlightenment scientists made the first attempts to conceptualize and empirically prove the existence of human races; in a second moment, a false and fixed conception of race was delineated; and finally, early paradigms were abandoned in favor of recognizing wide human variation with population genetics. Yet, this framework misses the fact there has never been a consensus on race definition. Another misrepresentation is facing it as just a false and old-fashioned idea. According to the author, the history of the idea is better understood if race is recognized as an epistemic tool whose imprecision is functional to oppression.

## 3 Discussion

### 3.1 Race, genes and intelligence controversy

Although the absence or presence of a soul were initially used to legitimize dehumanization, innate intelligence in its malleable definition and fine gradations gave ground to a stadial race classification, being much more functional to colonization purposes (7, 11).

Biological determinism of intelligence in the last century had various empirical focus, from craniometries to IQ tests. Heredity of intelligence research was fueled by prejudices and refueled them back, serving as theoretical support for eugenics and its measures of mass sterilizations, mass incarceration, bans on immigration, and finally genocide (12–14).

Since Galton, scientific sanctioned discourses on intelligence attributed socioeconomic status to biological traits in an attempt to naturalize socioeconomic inequality (15). When *The Bell Curve* reenacted the same leitmotiv at the end of last century, the economic grasp was that it offered *“evidence against both the premises of the welfare state in general and against the pro-equality social policies that grew out of the civil rights movement in particular”* (16). The book translated that capitalism resumed its focus on intelligence, resonating (as still does) informational economy and aligning with what Rose called the rise of neurogenetic determinism (17).

But, not long after that, in 2003, the controversy on IQ, race and gender seemed to have been settled once and for all after the Human Genome Project. It concluded that most genetic variations are within rather than between populations, meaning that racial groupings have no biological basis (18). But bad ideas die hard and scientific racism has an intermittent pattern (19). In a *Nature* hosted debate section on gender- or race-linked differences in intelligence in a Darwin 200 commemorative issue, Rose (20) argued that there was no reason to *“fan the flames of a dead debate”*. The author summarized main arguments against the “century old, but regularly recycled” debate. He pointed to the reification of a culturally variable conception of intelligence; he recalled that race is a highly unstable social construct that vastly varies over time and among different cultural contexts and has no biological foundation; and he challenged the possibility of biologically pinning different ways in which men and women think. Heritability was also recalled as a statistical concept, a measure only valid within-population, in a given environment, which does not state heredity causation. Therefore, further inquiry on gender and race inferiority fails valid scientific interest and is not justifiable, since it harms society (21).

Flynn (22), who gives name to the effect of generational rising of IQ scores attributed to environmental causes, disagreed. He argued that if Rose succeeds in convincing those against racism to not engage in debate, this could mean a *unilateral disarmament*. But Rose’s and Flynn’s were not the only positions. Meisenberg (23) stated that more research was relevant since the reasons for social inequities are unknown and genetics is a possible one. According to him, studies on the genetics of racial differences in intelligence are criticized because they threaten the belief in Mother Nature’s justice. Nonetheless, he continues, science and technology have reached a development stage in which they can finally act and, if granted access to data and conditions for research, *offending genes* can be corrected. The controversy was on again.

Oreskes (24), a pioneer in the studies of science denial, describes rhetorical strategies used to keep false controversies alive. First, someone with scientific credentials casts doubt on scientific consensus. Presented with doubt toward scientific evidence, scientists engage in showing more evidence. Yet, by doing so, they inadvertently give legitimacy to the controversy, conveying the impression that the consensus is debatable and there are two contrasting scientific options for the public’s choice. Denial of science does not equate to illiteracy, and tackling it demands identifying rhetorical strategies. Controversy and ignorance are socially reproduced, but scientific credentials are needed to legitimize it. The contender scientist usually “attacks the messenger” by falsely claiming that consensus was motivated not by science, but by political reasons. The next argument consists in attributing another possible cause to the phenomenon at stake. As the debate unfolds, when conceptual or methodological limitations are identified, challengers accuse those aligned with scientific consensus of being free-speech foes. If those strategies are successful, the controversy self-perpetrates on a repeated call for more research. Frequently keeping the question salient is the point, since doubt allows inaction.

Recent initiatives established National IQ datasets. However, Sear (25) argues that their primary data often came from small samples and that the mean IQ of many countries were estimated based on one sample and mostly unrepresentative of the national context. The author claims that most countries only had their estimation derived from evaluating children, which is problematic “*not only because these samples will be unrepresentative of the national population but also because psychometric test scores are affected by age*.”

On another front, Genome Wide Association Studies (GWAS) are used to study racial differences in intelligence. They are difficult to replicate (26) and even relying on large amounts of data, the search for correlations on the subject has yielded modest and non-consistent results (27, 28). The genetics mechanisms related to intelligence remain unclear (29). Social structure interferes with the distribution of genetic variation and its effects can surpass the expected length of genetic effects related to descent. For example, a study by Clark and Cummins (30) found that surname-basis prediction for education status lasted way longer than the expected influence of genetic variation related to family, persisting to 30 generations in some cases.

Richardson and Jones (31) argued that these studies have taken a lot of effort and funding, offering proportionally little results. Burt (32) additionally claimed that potential scientific rewards of applying recent Polygenic scores in social science research are not only outweighed by ethical and political risks, but by possible scientific costs of obscuring much more relevant structural disadvantages and cultural influences.

While controversy is kept alive, scientific publications claiming biological causes for differences in race and intelligence have been used to provide ground for weaponizing the criminal justice system against minorities and for restriction on immigration rules and welfare policies (33, 34).

### 3.2 Neurotechnology, intelligence and technocolonialism

Neurotechnology interventions based on racial or sexual differences in brain functioning are also presented as possible paths to combat injustice (35). Collecting vast amounts of genetic and neural data on racialized groups is described as a step to make science more just, representative and accessible.

Neural functions and structure are responsive to social and environmental stimuli. Thus, group differences in aging-related cognitive decline, for example, have been used for early detection purposes (36). It is well demonstrated that neural damage can be caused by malnutrition and stress due to social injustice (37, 38). Even though race is a social construct, it does not mean that it does not harm people’s health. Countering neurogenetic determinism is not a defense of post-racialism in medicine (39).

However, ‘neuralizing’ racism, even with the purpose of demonstrating brain effects caused by racism, remains ethically problematic. This is because neuroscience centers the individual in the understanding of a social, complex and interdisciplinary problem. If the intent is to combat racism, the risks seem to outweigh the potential benefit, since it is hard to imagine that neural images are the lacking evidence that would finally be effective in causing general empathy toward the suffering and death of marginalized groups. Naturalizing injustices means shifting responsibility from collective action to neurochemical functioning. By focusing on the brain level in neuroscience, it might appear tempting to target what is wrong in society in the brain (40). In addition, effectively tackling racism in brains would evidently demand more research, more data, and more time. In the case it is proven that racism causes an irreparable effect in brains, some would then cast doubt on the real cause of effects, whether they were natural or environmental, and so more research would be needed and the nature and environment racial differences’ controversy would begin all over again. Amidst all of this, there is the non-neglectable history of data gathered without authorization or used against the original purpose consented from racialized groups, with harmful and stereotype-reinforcing effects (41–43).

Race and intelligence are not the only malleable definitions. Neurotechnology in reality consists of a heterogeneous set of research, exams and interventions. Some of it has existed for more than 30 years, while others are currently non-existing but utopic projections of enhanced bodies and minds freed of any kind of mortal limitation. Neurotechnology can refer to both closed and open loop systems, meaning that they can just collect data on neural functioning, or be designed to monitor and automatically interfere in neural function. Some interventions are being developed with the intent of preventing or treating diseases and disorders recognized by science, while others are vaguely pursued to promote well-being or enhancement. Devices can be highly invasive or wearable. Finally, according to the context, the term may exclusively refer to direct interaction with the nervous system or include data collection and interventions that could infer from or interfere with neural functions.

When destined for neuroenhancement purposes, the malleable defined technology is designed to improve intelligence, another ill-defined construct. There are risks and when they are mentioned, arguments on the inevitability of scientific progress and the harm of preventing science to benefit people with disabilities commonly arise.

If discourses and enhancement practices are countered as divisive of human equality and based on Western ideals, then the malleability of the concept allows equating opposing enhancement to opposing treatment for disability. It is fully possible to be contrary to some purposes of neurotechnology interventions, but the umbrella-term is mobilized to give the impression that it is not.

False dichotomies are also a rhetorical strategy. Broadening or narrowing scope, changing focus, claiming that those who point out ethical risks are detrimental to human welfare, all of which is functional to avoid debating and addressing ethical challenges (44).

Even if the solution presented for the inequalities resulted from unequal access to neurotechnology is defending enhancement for all, the time this would take would be enough to collect a lot of data with hazardous effects. The case in which intervention occurs with a non-accessible cost (the most likely case-scenario), would increase inequity in educational and labor opportunities, and would further social and economic gaps, reinforcing colonial dynamics.

But, above all, neurotechnology shapes scientific, public and political imagination. There are a lot of assumptions involved when focusing on enhancing intelligence, including the definitions of intelligence and enhancement. Old conceptions of inferior and superior intelligence resurge, ones which were historically used to justify labor assignment and the “concert of nations.” Eugenics in the end led to genocide, but it gave scientific grounds for naturalized class structure and social inequities much earlier. In order to present neurotechnology as a means to combat injustice, injustice is naturalized. Mastering nature for social purposes is part of a known relationship pattern between science and nature in colonialism.

Neurotechnology is part of a technological convergence with genetic editing and artificial intelligence, in which AI discourses also contribute to shape the highly malleable definition of intelligence, openly arguing for its revision. The tactic is working well and the equivalence between cognitive ability and the ability to realize human activities of economic worth is gaining traction (45). Taking this last definition trend into account when surveillance and neuroenhancement are applied to economic purposes, they not only increase risks to privacy and consent in the highly coercive laboral context, but there also exists the possibility of reinforcing colonial perspectives of wealth as an indelible manifestation of biology. Naturalization of job assignments and of inequality of opportunity according to measures of intelligence was also at the core of eugenics. Hegemony of neuroenhancement imagination represents a colonial risk to Indigenous Peoples and to any other groups of people who do not share the same cosmovision, and cannot or do not want to be subject to such interventions. Scientific legitimized hierarchy of superior and inferior groups of humans is an essential part of colonial dynamics.

## 4 Conclusion

Neurotechnology is a very encompassing term, and while research and development of devices and techniques destined to restate physiological functions lost by disorders or diseases is commendable, enhancing minds and equating intelligence to economic worth is less so.

Concepts, techniques and devices related to neurotechnology, mainly those related to intelligence, might be used to reinforce colonial hierarchies. Equating intelligence with economic worth and productivity is not a universal perspective, and technological development informed by this logic is threatening to the non-Western praxis of living and knowing. The assumption that intelligence variability between groups is biological/genetic in its origin has historically justified hierarchization, dehumanization, domination and exploitation of gender, race and class.

Unequal access is not the only or even the main ethical challenge concerning neurotechnological advancements in the Global South. Future applications of neurotechnology should be guided by the value of epistemic justice and driven by beneficiary participation in all technological development phases. Given the constant reissuing of scientific racism, a path toward collective construction of non-colonial ethical and legal approaches to neurotechnology involves promoting literacy on previous biological determinism theories and their rhetorical strategies for the public in general, for representatives of historically marginalized groups, and for scientists.

## Data Availability

The original contributions presented in the study are included in the article/supplementary material, further inquiries can be directed to the corresponding author.
